# Temporal Expectation and Attention Jointly Modulate Auditory Oscillatory Activity in the Beta Band

**DOI:** 10.1371/journal.pone.0120288

**Published:** 2015-03-23

**Authors:** Ana Todorovic, Jan-Mathijs Schoffelen, Freek van Ede, Eric Maris, Floris P. de Lange

**Affiliations:** 1 Donders Institute for Brain, Cognition and Behaviour, Radboud University Nijmegen, 6500, HB Nijmegen, The Netherlands; 2 Max Planck Institute for Psycholinguistics, Radboud University Nijmegen, 6500, HB Nijmegen, The Netherlands; Kyoto University, JAPAN

## Abstract

The neural response to a stimulus is influenced by endogenous factors such as expectation and attention. Current research suggests that expectation and attention exert their effects in opposite directions, where expectation decreases neural activity in sensory areas, while attention increases it. However, expectation and attention are usually studied either in isolation or confounded with each other. A recent study suggests that expectation and attention may act jointly on sensory processing, by increasing the neural response to expected events when they are attended, but decreasing it when they are unattended. Here we test this hypothesis in an auditory temporal cueing paradigm using magnetoencephalography in humans. In our study participants attended to, or away from, tones that could arrive at expected or unexpected moments. We found a decrease in auditory beta band synchrony to expected (versus unexpected) tones if they were unattended, but no difference if they were attended. Modulations in beta power were already evident prior to the expected onset times of the tones. These findings suggest that expectation and attention jointly modulate sensory processing.

## Introduction

In a world where the senses are continuously stimulated, perception acts to optimize information processing by prioritizing based on behavioral goals and expectations. This prioritization can encompass spatial locations or object features, but the brain can also prioritize temporal windows. There may be two distinct classes of processes guiding temporal selection: *temporal expectation*, the ability to extract temporal regularities from the environment, and *temporal attention*, the state of anticipating relevant future events. Researchers who focus on temporal expectation typically find that temporally predictable events lead to *less* neural activity than temporally unpredictable events [1–4]. Conversely, researchers who focus on temporal attention typically find that attended events lead to *more* neural activity than unattended events [5, 6]. This would suggest that expectation and attention act as opposing forces in sensory processing. However, expectation and attention are largely studied either in isolation [7–9] or conflated [10]. For example, temporal cueing studies often contrast expected, attended events with unexpected, unattended ones [11, 12], making it difficult to tease apart the contributions of expectation and attention [13, 14].

Several studies have looked into the interplay of expectation and attention on sensory processing, but the picture that emerges is ambiguous. On the one hand, it has been proposed that attention is necessary for expectations to affect sensory processing [15]. On the other hand, mismatch negativity studies, which compare rare deviant events to frequent standards, suggest that attention is not necessary for expectations to form [16]; although see [17]. Finally, a spatial cueing study where expectation and attention were orthogonally manipulated observed an interaction in the form of expectation suppression for unattended events, but expectation enhancement for attended events [18]. This result is consistent with the suggestion that expectation interacts with attention in a synergistic manner [19, 20], with increased activity to attended events and attenuated activity to ignored events. Here we test whether this interaction can be generalized to auditory temporal expectation.

We orthogonally manipulated expectation and attention in an auditory temporal cueing paradigm while recording neural activity using magnetoencephalography (MEG). Participants listened to pairs of tones which were separated by a predictable or unpredictable temporal interval (leading them to form a focal or distributed temporal expectation), while performing a task on either the first or the second tone in each pair. We found evidence for interacting effects of expectation and attention, with a decrease in auditory beta power when having focal (versus distributed) expectation, but only if attention was drawn away from the tones. With attention, there was no effect of temporal expectation. This suggests that expectation and attention jointly act to guide sensory processing, with expectation potentially facilitating the filtering out of temporally predictable, irrelevant events.

## Methods

### Participants

Twenty five healthy participants (17 female, age 23.7 + 7.8 years, mean + SD) took part in the experiment upon signing an informed consent form in accordance with the Declaration of Helsinki. All participants had normal hearing and no history of neurological or psychiatric disorders. Sixteen of these participants also took part in another auditory expectation study on the same day (reported in [29]). The dataset of one participant was not analyzed due to excessive artifacts (>30% of the trials). The study was approved by the regional ethics committee (Committee on Research Involving Human Subjects, Region Arnhem-Nijmegen, The Netherlands).

### Stimuli and experimental design

The experimental stimuli consisted of brief pure tones (frequency 1000 Hz or 1200 Hz, duration 5 ms, ~70 dB SPL), which were presented binaurally via MEG-compatible air tubes. Stimuli were presented using a PC running Presentation software (Neurobehavioral Systems).

Each trial started with the presentation of a central fixation cross (2–4s). A standard tone (1000 Hz) was then presented twice in quick succession, with one of five possible inter-stimulus-intervals (ISI) between the two presentations (250, 375, 500, 625, and 750 ms). The fixation cross remained present for an additional period (0.5–1 s), followed by a short period when it was removed, in which the participants could freely move their eyes and blink (1.5–2 s). This resulted in a 4–6 s intertrial interval, defined as the interval between the last tone of the current trial and the first tone of the next trial. Participants were instructed to listen to the tones and press a button with their right index finger if they heard a deviant tone (1200 Hz). Each block consisted of 91% trials with standard tones and 9% trials with a deviant tone.

**Fig 1:**
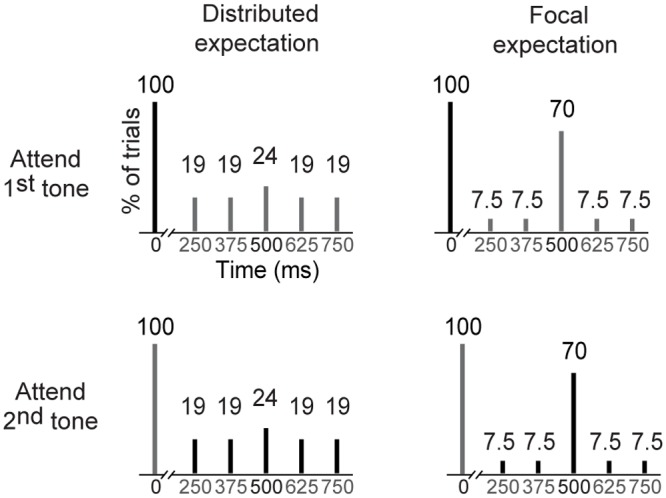
Task structure. All trials contained two tones. The first tone is shown here appearing at time point zero. In blocks with a distributed temporal expectation (left panels), the second tone appeared at a similar rate at five possible ISIs after the first tone, while in blocks with a focal expectation (right panels) the middle ISI was most frequent. Attention was either on the first tone (top panels) or the second tone (bottom panels). Numbers above the bars represent the percent of trials per block in which standard tones appeared at their respective time points. Numbers on the x-axis denote the duration of the ISIs. Only tone pairs with an ISI of 500 ms were analyzed.

We manipulated the temporal *expectation* of the second tone by varying the relative frequencies of the different inter-stimulus intervals per block (Fig. 1). Recent research has shown that listeners are sensitive to the distribution of tone frequencies, with narrower distributions leading to a stronger expectation that the following tone will fall close to the mean [21]. We adapted this paradigm to the temporal domain, by creating blocks with narrow or wide temporal distributions. In blocks with a wide temporal distribution, the second tone appeared at all five ISIs with similar rates (19%, 19%, 24%, 19%, 19%). This led to a state of *distributed* temporal expectation, as the second tone could be roughly equally expected to appear after any of the five possible intervals. Conversely, in blocks with a narrow temporal distribution, the second tone appeared at the middle ISI frequently in comparison with the surrounding four ISIs (7.5%, 7.5%, 70%, 7.5%, 7.5%). This led to a state of *focal* temporal expectation, as the second tone was most likely to appear after the duration of the middle temporal interval. We were interested in comparing neural activity elicited by tones at this middle ISI in relation to temporal expectation. The tone pairs separated by the remaining four ISIs were not analyzed due to a small number of trials. In sum, each of the analyzed trials contains two appearances of a 1000 Hz tone, separated by 500 ms, where the temporal predictability of the second tone varied per experimental block. Given that tones at the middle ISI were also comparatively rare in the condition of distributed expectation (as a logical consequence of our experimental design), we doubled the amount of blocks within this context to generate a sufficient amount of trials for statistical analysis. In total, there were four blocks with distributed temporal expectation (88 tones at the middle ISI and 254 tones at the remaining ISIs), and two blocks with focal temporal expectation (120 tones at the middle ISI and 52 tones at the remaining ISIs). When the trials with deviant tones and artifacts were removed, an average of 76 trials with distributed temporal expectation and 112 trials with focal expectation were analyzed per subject. Block order was counterbalanced across subjects.

In addition to temporal expectation, we also manipulated temporal *attention*, in an orthogonal fashion. We presented the task-relevant deviant tones on either the first or the second position in the tone pairs, but this position was held constant per block. In other words, in every block participants were informed whether to expect targets to appear only on the first or only on the second tone position. This manipulation thus drew their attention to the moment the first (or second) tone was displayed, and made the moment of presentation of the other tone task-irrelevant.

Prior to the experiment, there was an 8 min practice session which contained 60 trials. The distribution of trial and block types in the practice session was identical to the distribution in the experiment. The experiment lasted one hour.

### MEG measurements

Brain activity was recorded using a whole-head MEG with 275 axial gradiometers (VSM/CTF Systems) in a magnetically shielded room. Head localization was monitored continuously during the experiment using coils placed at the nasion and left and right ear canals. As an aid for eye blink and heartbeat artifact rejection, an electro-oculogram (EOG) and electrocardiogram (ECG) were recorded, using 10-mm-diameter Ag—AgCl surface electrodes. For the EOG, these electrodes were placed on the supraorbital and infraorbital ridge of the left eye. For the ECG, these electrodes were placed above the clavicle on the left side, and on the abdomen on the right side, ~15cm below the rib cage.

### MEG data analysis

The data were analyzed in Matlab (MathWorks) using the FieldTrip toolbox developed at The Donders Institute for Brain, Cognition, and Behavior [22]. Data analysis was performed on the trials consisting of standard tones only where the response was correctly withheld, and which were temporally separated by 500 ms. Data epochs of interest were checked for artifacts caused by muscle activity and SQUID jumps. We used semiautomatic routines to discarded contaminated trials. Subsequently, independent component analysis [23] was used to remove any remaining variance attributable to eye blinks and heartbeat artifacts [24]. Finally, the data were visually inspected and any remaining trials with artifacts were removed manually.

### Time—frequency analysis

We calculated time—frequency representations (TFRs) using a Fourier transform approach applied to short sliding time windows. For the low frequencies (5–35 Hz) the length of the time window T was frequency dependent, and consisted of three oscillation cycles for each frequency (Δ*T* = 3/*f*). We applied a Hanning taper to control for spectral leakage. Effectively, this led to an adaptive frequency resolution of Δf = 1/T = f/3. The time windows were advanced in steps of 25 ms. For the high frequencies (40–120 Hz), we used a fixed window length of 200 ms with a Δf = 20 Hz frequency smoothing, using multitapers [25]. Power estimates were first calculated separately for the horizontal and vertical component of the planar gradient and then summed [26]. This simplifies the interpretation of the sensor-level data because it places the maximal signal above the source [27]. Raw planar gradient power estimates were converted to decibel (dB), by log-transforming with base 10 and then multiplying by 10. We then subtracted the power in the baseline window, centered at 500 to 400 ms before the presentation of the first tone. The TFR was calculated and baseline corrected per trial, a procedure that is equivalent to taking a relative baseline of raw power. We then selected the median of the planar gradient power estimates across trials within each condition. Selecting the median, rather than the mean, has the benefit of the data being less affected by noise and outliers. This is especially relevant for power estimations which are always positive, i.e. noise and outliers will sum rather than average out if the mean is used. The obtained TFRs consist of both evoked and oscillatory activity, and do not allow for a decisive separation of the two. We additionally used a multitaper approach [28] to visualize the beta power in the pre- and post-stimulus time windows where we found clusters of significant differences between conditions, by estimating the spectral energy at the peak frequency of observed differences (23 Hz) with a 9 Hz frequency smoothing bandwidth. Furthermore, we conducted a within-subjects trial-by-trial analysis, in which we separately calculated TFRs for the theta (5–7 Hz) and beta (13–32 Hz) bands, in two time windows corresponding to pre- and post-stimulus activity. These calculations were done separately (beta using an early, pre-stimulus window, theta using a post-stimulus window) in order to prevent temporal uncertainty in the power estimates from influencing the results of the correlation analysis. The range of frequencies within the beta band was chosen to match the range where we found an interaction between temporal prediction and attention in the between-subjects analysis. We previously observed that differences in low frequency power across expectation conditions correspond to effects obtained with ERFs [29], when using an equally long tone of the same frequency as used in this experiment. This is a consequence of the latency of the evoked response to auditory tones, which peaks about 100 ms post-stimulus and decreases over the next 100 ms [27], thus corresponding to a single 5 Hz cycle. The post-stimulus theta band thus contains the spectral energy that is produced by the evoked responses.

### Source localization

We acquired structural MRI scans of 23 out of 24 participants in our study using a 1.5 T Siemens Magnetom Sonata system (Erlangen, Germany). Three of those scans were of insufficient quality for source reconstruction. We thus performed source reconstruction on 20 participants using a subject specific realistic head model extracted from the individual segmented MRIs. For the remaining four participants we used a head model created from a template MRI. We identified sources of activity using a frequency-domain beamformer, Dynamic Imaging of Coherent Sources (DICS). The brain volume of each participant was discretized to a grid with a 1 cm resolution and the lead field matrix was calculated for each grid point using a single shell volume conduction model based on the inner surface of the skull. For each grid position we computed an adaptive spatial filter to estimate oscillatory power in the entire brain [30, 31], using the lead field matrix at that location and a cross-spectral density matrix defined between all pairs of MEG sensors. We used a ‘common filter’ approach, where for a given contrast of interest the spatial filter was computed from a cross-spectral density matrix that had been estimated from the data of the baseline and post-stimulus period combined. We computed oscillatory activity in several frequency bands and time windows. For each of these power estimations, the baseline window was equally long as the post-stimulus window of interest, beginning at 500 ms prior to the onset of the first tone in a trial. First, in order to examine the average sensory representation that the tones elicited, we estimated theta power (5–7 Hz) in the 300 ms after the first tone in all trials by means of the Fourier transform of the Hanning-tapered signal. Second, in order to examine the spatial specificity of the experimental effect that we obtained at the sensor level, we calculated beta power in a pre-stimulus window 320 to 100 ms prior to the second tone, and in a window related to stimulus processing, from 20 ms prior to 130 ms after the onset of the second tone. These two time windows correspond to the first two clusters obtained in the statistical tests. We then made an averaged source reconstruction over these two time windows in order to get an estimation of the source of the experimental effect. Here we applied a multitaper approach [28] to obtain a spectral estimate at 23 Hz with a 9 Hz frequency smoothing bandwidth, resulting in a power estimate from 14 to 32 Hz. We compared different conditions by subtracting the power values at each grid point. The individual subjects’ source reconstructions were coregistered to their anatomical MRIs, and the anatomical and functional data were subsequently spatially normalized to the International Consortium for Brain Mapping template (Montreal Neurological Institute, Montreal, Quebec, Canada; http://www.bic.mni.mcgill.ca/brainweb). After spatial normalization, the source reconstructions were averaged across subjects.

### Statistical analysis

Oscillatory activity in different conditions was statistically compared using nonparametric cluster-based permutation tests [32]. This type of test controls the type I error rate in the context of multiple comparisons by identifying clusters of significant differences over space, time, and/or frequency instead of performing a separate test for all pairs of sensors, samples, and frequency bins. All cluster-level statistics, defined as the sum of *t* values within each cluster, were evaluated under the permutation distribution of the maximum (minimum) cluster level statistic. This permutation distribution was approximated by drawing 5000 random permutations of the observed data. The obtained *p* values represent the probability under the null hypothesis (full exchangeability, i.e. no difference between the conditions) of observing a maximum cluster-level statistic that is larger than the observed cluster-level statistics, and a minimum cluster-level statistic that is smaller than the observed cluster-level statistics.

In order to examine auditory activity, we averaged over the spatial (channel) dimension on the basis of independent localization of the 10 left and 10 right channels that showed the most robust tone-related activity. We estimated the sensors based on the average activity of all the tones in our experiment, irrespective of experimental condition. We then assessed whether there were significant spectrotemporal clusters of differential activity between the experimental conditions, within a 1-second period after the first tone, in either low (5–35 Hz in steps of 1 Hz) or high (40–120 Hz in steps of 3 Hz) frequencies. For assessing the interaction between expectation and attention, we applied cluster-based permutation tests that evaluated whether there was a difference between the two levels of attention (task-relevant versus task-irrelevant) with respect to the expectation effect (distributed versus focal). To qualify the nature of the interaction, we constrained the analyses to the time points and the frequency bands of three clusters where we observed a significant interaction. We conducted T-tests for these post-hoc analyses. The p-values reported here are part of a post-hoc analysis of interaction effect identified in three time-frequency windows (using a method that controls the false rate across time and frequency). Because these time-frequency windows were identified on the basis of the interaction effect in the data, the reported p-values of this post-hoc analysis can be used as an index that allows us to explore the nature of the interaction effect (of which the existence was demonstrated in an analysis that controls the false alarm rate). Furthermore, to determine whether the effect which was observed before the onset of the second tone, had an influence on subsequent stimulus processing of this tone, we conducted a trial-by-trial analysis in which we correlated pre-stimulus beta power (13–32 Hz) with post-stimulus theta power (5–7 Hz) for each individual subject. We performed a T-test to assess whether the obtained correlation coefficients differed significantly from zero at the group level.

## Results

### Behavioral results

Participants were instructed to press a button whenever they heard a deviant tone. They did this task with high accuracy, correctly responding to 95.6% of the deviants (SD = 5.3), and correctly withholding a response to 98.8% of the standards (SD = 0.5). It is important to note that deviant tones in this experiment were rare, and that we only analyzed reaction times to deviant tones occurring at the middle ISI (a total of 14 trials). The behavioural results on the deviants should therefore not be taken as a strong indicator of the presence or absence of experimental effects on the standard tones, which were the subject of our MEG analyses. Participants were faster to respond to the deviants when they occurred on the second tone as opposed to the first tone (first: RT = 844 ms, second: RT = 635 ms; F(1,23) = 46.3, p<0.01). Reaction time did not depend on whether temporal expectation was focal or distributed (F(1,23) = 3.51, p = 0.56), nor was there an interaction with the locus of attention (first versus second tone: F(1,23) = 2.55, p = 0.12).

### Neural activity modulations elicited by auditory processing

**Fig 2:**
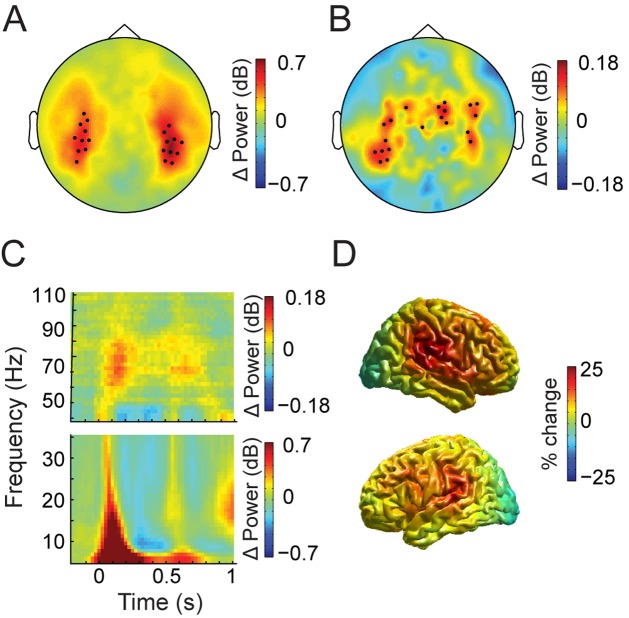
Localization of auditory activation. ***A,*** Topographic representation of MEG channel activation for all tones at 5–35 Hz in the interval of maximal activity after tone onset. The maximally activated channels in each hemisphere are highlighted. ***B,*** Same as **A** for frequencies from 50 to 90 Hz. ***C,*** Time-frequency representation for all analyzed trials, averaged over the selected sensors. ***D,*** Source reconstruction of low frequency power in the theta band (5–7 Hz) shows bilateral peaks of activation in superior temporal cortices.

Tones reliably elicited bilateral activity over temporal sensors. We calculated tone-related activity for all the tones regardless of experimental condition, and compared their spectral power to baseline activity. In the low frequencies (5–35 Hz), neural activity to the average of all tones was maximal 50–150 ms following tone onset, while in the high frequencies (50–90 Hz) this activity was maximal after 100–200 ms. From these time windows, we selected 10 left and 10 right channels which showed strongest activity (Fig. 2A,B). The spectral signature of low frequency neural activity over all trial types in these channels (Fig. 2C) was predominantly visible as a power change in the theta frequency band (5–7 Hz), which spread to the alpha (8–12 Hz), and beta bands (15–25 Hz). Changes in gamma band power (50–90 Hz) following tone presentation were evident as well. In order to confirm that low frequency power changes involved auditory activity, we also performed source reconstruction of theta-band activity of these tones, which showed a focal increase in power over temporal cortices (Fig. 2D), consistent with a source in the auditory cortex.

### Interaction effects on beta-band oscillatory activity between temporal expectation and attention

**Fig 3:**
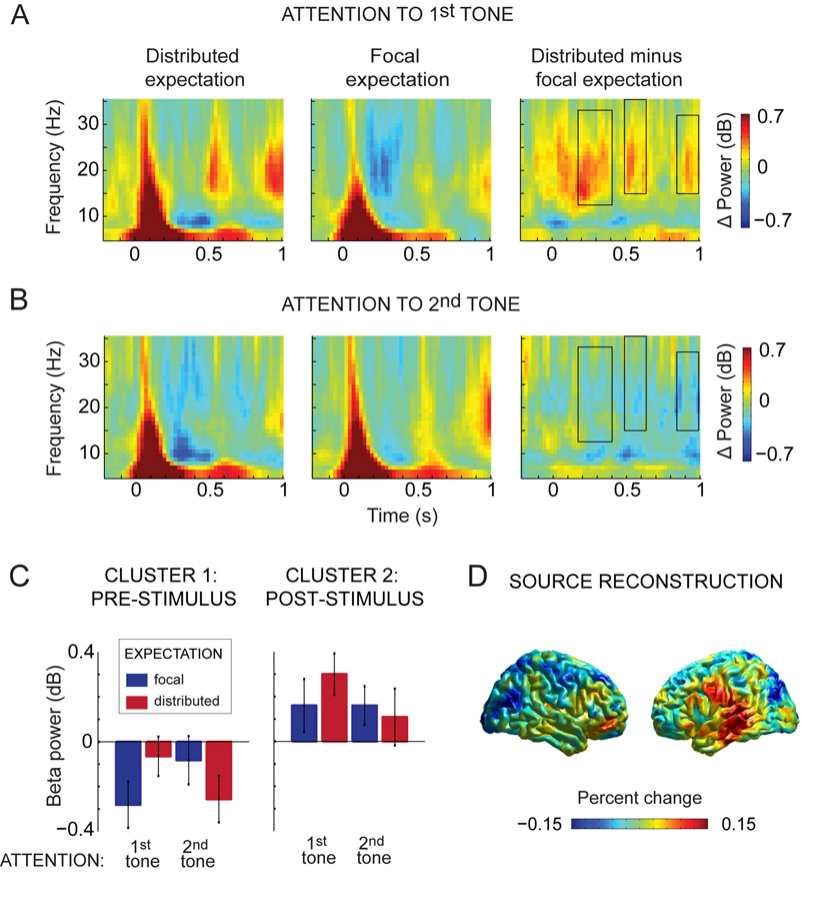
Time-frequency representations of stimulus-related activity in selected sensors as a function of expectation and attention. ***A,*** When participants were attending to the first tone in each tone pair, there was more beta band activity in trials where temporal expectation was distributed (left panel) than when it was focal (middle). Rectangles represent spectrotemporal clusters where a significant interaction of expectation and attention was found (right). ***B,*** When participants were attending to the second tone in each tone pair, there was no difference in beta band activity in trials where temporal expectation was distributed (left panel) than when it was focal (middle). Rectangles represent spectrotemporal clusters where a significant interaction of expectation and attention was found (right). ***C,*** Interaction between expectation and attention during pre-stimulus activity (the leftmost cluster in ***A*** and ***B)*** and post-stimulus activity (the middle cluster). ***D,*** Source reconstruction of the beta-band interaction between temporal expectation and attention.

We first investigated whether there were overall differences in oscillatory activity for distributed vs. focal expectation, as well as for attention to the first or to the second tone. Somewhat surprisingly, neither of these factors modulated the spectral signature of auditory processing in isolation (expectation, low frequencies: p = 0.164, high frequencies, p = 0.290; attention, low frequencies: p = 0.164, high frequencies: p = 0.582). However, an interaction between temporal expectation and temporal attention was evident in the beta band (p = 0.019), beginning prior to the onset of the second tone and lasting throughout the trial epoch. This interaction was most prominent in three clusters: before the onset of the second tone (360–100 ms pre-stimulus, frequencies: 13–32 Hz, Fig. 3C—left panel), during early tone processing (20 ms prior to tone onset to 130 ms after tone onset, frequencies: 15–29 Hz, Fig. 3C—right panel), and during late tone processing (350–500 ms after the onset of the second tone, frequencies: 15–35 Hz). We found no interaction between our experimental factors on oscillatory activity in the high frequency range (p = 0.177). We further investigated the nature of the low frequency interaction by comparing average beta power in these three time windows between conditions of distributed and temporal expectation, separately for each of the two attention conditions. We found that the effect of temporal expectation on beta power depended on whether a tone was attended or not. When the first tone was attended (Fig. 3A), there was *less* beta-band activity when expectation was focal than when expectation was distributed. This was true both prior to the onset of the second tone (t(23) = 2.91, p = 0.007) as well as after its onset (t(23) = 2.76, p = 0.011). This expectation effect was absent when the second tone was attended (pre-stimulus: t(23) = -0.96, p = 0.348; post-stimulus: t(23) = -1.42, p = 0.17), with even a hint of *more* beta-band activity when expectation was focal than when it was distributed (Fig. 3B). During late tone processing we found no difference in expectation conditions (unattended: t(23) = 1.75, p = 0.09, attended: t(23) = -1.54, p = 0.136).

To gain more specificity with respect to the source of the interaction between temporal expectation and temporal attention, we used a beamformer approach to localize beta power in the time windows corresponding to the pre-stimulus and post-stimulus effects (i.e. the first two clusters). We averaged this source-level activity over the pre-stimulus and post-stimulus time windows. We compared the temporal expectation effects by calculating the difference in average power between brain activity related to distributed vs. focal expectation separately for each attention condition, and then subtracted the expectation effect when the first tone was attended, from the expectation effect when the second tone was attended. This procedure is equivalent to the sensor-level analysis, and the interaction we observed at the sensor level should manifest as increase in beta power in the source plot. We found that the interaction between temporal expectation and attention was most prominent over left superior temporal cortex (Fig. 3D), which is in line with a sensory effect. There was an additional positivity in the right frontal cortex, a source which has often been described in mismatch negativity studies [33]. Notably, the negativity in the source plots, spanning large areas of the occipital and right parietal cortex, is opposite in direction from our sensor level effect, indicating that these areas also potentially involve a beta modulation, but that this modulation is opposite in sign compared to the significant interaction we observed in the sensors above auditory cortex.

### Beta activity preceding a tone is predictive of activity later elicited by that tone

Finally, we were interested to know whether activity in the beta band prior to the onset of the second tone, which we found to be jointly modulated by temporal expectation and temporal attention, is predictive of the neural activity that was elicited by the second tone. To investigate the relationship with pre-stimulus activity, we correlated pre-stimulus beta power (which we found to be related to expectation and attention), with theta power (which contains the energy of the evoked response) following the onset of the second tone. To control for factors which might lead to correlated power in all frequency bands within a trial (e.g. head movement, noise, power fluctuation in lower frequencies, etc.) we removed all common variance between pre-stimulus beta power (360–100 ms pre-tone 2, 13–32 Hz) and pre-stimulus theta power (360–100 ms pre-tone 2, 5–7 Hz) prior to the correlation with post-stimulus theta power (0–300 ms post-tone 2, 5–7 Hz). We found that the magnitude of pre-stimulus beta power was positively correlated with the magnitude of the post-stimulus activity after correcting for instantaneous correlations (median r = 0.12, t(23) = 8.67, p<0.001). In other words, a stronger beta power prior to tone onset was associated with a stronger neural response to the tone after its onset.

## Discussion

In this study, participants listened to pairs of tones while performing a task either on the first or on the second tone, where the expectation of the onset time of the second tone was either distributed or focal. We observed that temporal expectation and temporal attention jointly modulated beta band activity in an interacting fashion. We found that expectation led to a decrease in beta power, but only in the absence of attention (Fig. 3A). With attention, an expectation effect was not significantly present (Fig. 3B). This interaction was most pronounced in left superior temporal cortex, suggestive of a localization in auditory cortex. Additionally, we found that higher beta power prior to tone onset led to higher neural activity (theta power) evoked by the tones. Our results suggest that expectation guides temporal attention: expectation attenuates the response to events happening at unattended moments, but it does not affect the response during attended time windows.

Temporal expectation and attention guide perception by highlighting moments in time when sensory processing becomes particularly efficient. For example, stimuli are recognized more quickly if their appearance is temporally predictable [6, 34, 35]. While temporal processing involves several specialized cortical circuits, such as the basal ganglia or the cerebellum [36], processing of time is partly also local to sensory cortices [37]. This local processing may rely on oscillatory processes that entrain to rhythms present in external stimulation [6, 38–40]. Sensory beta band activity is known to be modulated by integrative cortical processes such as attention or decision making [41, 42], and beta oscillations are suggested to play a role in conveying predictions about future sensory input [43]. For example, beta band activity has been linked to novelty processing in the auditory system in humans [44], with increased power to unexpected tones in a mismatch negativity (MMN) paradigm. Additionally, beta power is predictive of auditory repetition suppression [45], a phenomenon that has strong links with stimulus expectation [29, 46]. In studies of temporal attention, low frequency entrainment to externally generated rhythms [6] can become coupled to modulations in beta power [47, 48] which varies as a function of stimulus anticipation [49–52]. Interestingly, our results showed a similar pattern of beta power modulations, but in the absence of an external rhythm. Although the task did not involve estimating durations, participants could make use of the temporal structure of the sounds in order to enhance their detection performance. Each of the trials contained only two tones, where the temporal uncertainty of the second tone was derived based on the distribution of inter-stimulus intervals across trials within each block, while the inter-trial interval varied randomly. While listening to the tones, beta band activity increased or decreased as a joint function of expectation and attention. How did this beta modulation, which is typical for rhythmic stimulation, arise in a situation where there is no rhythm? One possibility is that assessments of interval durations rely on oscillatory processes, and that already one tone may be sufficient to entrain activity, perhaps via a phase reset, in the presence of a task that benefits from accurate time assessment. The modulation in beta power may also reflect an endogenous change in neural synchrony related to expectation and attention. Finally, this modulation could reflect entrainment to activity related to omitted tones. In the trials we analyzed, there were two shorter and two longer intervals when the second tone could have potentially occurred, but did not. Unexpected auditory omissions have previously been shown to result in activity in the auditory cortex [53, 54]. This raises the intriguing possibility that not only a rhythmic stimulus train, but also a rhythmic *expectation* of a stimulus, could underlie oscillatory activity related to temporal processing. However, neither of these hypotheses is sufficient to explain why the experimental effects were particularly prominent in the blocks where participants performed a task on the first tone in the pairs. In these blocks, once the trial started with the presentation of the first tone, there was no longer anticipation that a target tone might appear on the second tone. A more accurate assessment of the interval between the two tones would thus not be beneficial for performing the task. In fact, this would have led to a main effect of temporal attention, which we did not observe. Similarly, we have previously observed that activity elicited by tone omissions scales with the (im)probability of the omission [29]. As the absence of tones at early ISIs was more probable when temporal expectation was focal than when it was distributed, beta band modulations during tone omissions would also be expected to be more prominent in the distributed relative to the focal condition, which would have been reflected in a main effect of stimulus expectation. However, we did not find such an effect either. Instead, we found an interacting effect of expectation and attention. It is possible that the manipulations of attention and expectation in the study were weak, and thus did not lead to main effects: the task could be solved without much attention thereby precluding a strong attentional modulation in our subjects. At the same time, the most likely tone was on the middle ISI in both types of expectation conditions, precluding a strong expectation modulation. However, the presence of the interaction indicates that both temporal expectation and temporal attention played a role in tone processing. An alternative possibility is that the main effects are averaged out because expectation differently modulates auditory activity in the presence of attention than in its absence, by filtering out potentially distracting information if it is predictable but preserving information if it is task-relevant.

Auditory processing appears to be especially sensitive to temporal information [55]. Temporal processing is inextricably linked to both stimulus expectation (reflected in the adjustment of neural activity to temporal regularities in the environment) and stimulus attention (reflected in the anticipation of a stimulus in order to assess it). In many situations, these two top-down factors cannot be well separated. For example, rhythmic stimulation prompts temporal attention around the occurrence of stimuli, but later beats are more temporally predictable than early ones, based on the stimulus history. Expectation and attention are also often conflated in research, especially in Posner-type tasks where stimuli appear in validly cued temporal windows more often than in invalidly cued ones. However, when attention is held constant, temporal expectation is found to reduce auditory activity [56, 57], and when expectation is held constant, temporal attention increases it [35]. This raises the question whether expectation and attention represent opposing forces, with the potential to cancel each other out. A recent review of predictive auditory processing suggests that unexpected events may lead to increased neural activity when unattended, but decreased activity when attended [2]. Evidence from the visual domain supports this suggestion, as a spatial attention study found increased V1 activity to unexpected gratings if they were unattended, but reduced activity if they were attended [18]. We hypothesized that joint contributions of temporal expectation and temporal attention represent a general principle of sensory processing: when it is possible to form an expectation, an attended stimulus may undergo enhanced processing, but an unattended stimulus will be suppressed. We indeed found an interaction between expectation and attention. This interaction involved a modulation of beta power, and was already evident prior to the occurrence of the tones. However, once attention was directed to the time window of interest, expectation did not modulate auditory beta power. This might also indicate that temporal attention is relatively imprecise in comparison with the inter-stimulus intervals in our study, leading to prioritized processing around the entire time when the second tone may occur but without the capacity for further temporal fine-tuning based on expectation. Furthermore, the onset of the second tone overlaps in time with late processing of the first tone. The observed activity differences could thus also be partly related to late processing of the first tone, rather than to a preparatory process related to the second tone. Namely, additional attention might have been allocated to late processing of the first tone when the temporal prediction of the second tone was more precise, because the first tone is more informative about the onset of the second tone in this condition. However, in this case, the expected result would be a difference between focal and distributed processing when the second tone is task-relevant, whereas we found a difference only when the first tone was task-relevant.

It has been suggested that sensory correlates of timing involve activity generated by the motor system [58]. Even though the cortical sources of the beta band activity that we observed were predominantly located over auditory cortex (Fig. 3D), it cannot be excluded that they may also be partly generated in the motor system. However, none of the analyzed trials contains an overt motor response, and we did not observe reaction time differences across the different expectation blocks, which would have signaled a difference in motor preparation. Additionally, pre-stimulus modulations of beta power tend to be opposite in direction of post-stimulus motor activity [59]. In our study, neural activity elicited by the tones is indexed by theta activity (which, rather than being a true oscillation, represents a frequency description of the evoked field). Instead of a negative relationship, we found a positive one: within subjects, on trials where beta power was higher prior to the onset of the second tone, neural activity elicited by the tone was also higher. This effect was however not consistent enough to translate into a theta modulation by expectation or attention across subjects. Interestingly, a modulation of beta power in the opposite direction of the one we observed in our sensor level analyses was visible over parietal cortex, which is similar to results of a recent study where participants judged whether a tone was delayed relative to the beat consisting of previous tones [47]. This study found that higher beta power in auditory cortex indicated more precise temporal judgments. At the same time, lower beta power in motor cortex also indexed better task performance. Our study confirmed that temporal expectation and temporal attention lead to modulations of beta power in auditory cortex. We additionally show that the joint effect of expectation and attention is an interacting one, with expectation decreasing beta power in the absence of attention, but not modulating it in its presence.

Predictive coding models posit that unexpected events lead to more neural activity than expected ones [60, 61]. These models suggest that the brain acts as a probabilistic inference machine [20], continuously forming predictions about future input. Numerous recent studies have provided evidence for this account, in auditory processing [1, 3, 8, 9, 62, 63], as well as visual processing [64–66] and somatosensory processing [51, 67]. In our paradigm, the occurrence of the first tone was temporally unpredictable, but once it was perceived, an expectation could be formed about the onset time of the second tone. We observed an attenuation of beta power preceding and following tones with a more predictable onset time. Lower beta power to the occurrence of the second tone when expectation was focal (i.e. more predictable) could thus be indicative of reduced prediction error. Interestingly, we only evidenced a decrease in beta power prior to focally expected tones when attention was drawn *away* from the tones. Once attention was already directed *towards* the time window when the second tone was about to appear, temporal expectation did not additionally modulate neural activity. This finding would not directly follow from predictive coding models, but might be in line with the suggestion that the role of expectation is to monitor contingencies in the world and to direct attention towards unexpected events [68, 69].

At first sight, the predictive coding account, which posits a decrease in neural activity to expected events, and the attentional cueing account, which posits an increase in neural activity to expected/attended events, appear to be at odds with each other. Predictive coding models [60] assume the existence of two types of neural units: those that form a sensory representation of the stimulus (representational neurons), and those that compute the mismatch between the incoming representation and the predicted one (error neurons). The larger response to surprising events stems from larger error neuron activity to the mismatch, which leads to a larger adjustment of the sensory representation. It has been shown, however, that predictive coding models can also successfully simulate attentional enhancement in Posner-type paradigms, by modulating the gain of the representation units [70, 71] or error units [19] in the presence of attention. This has led to the suggestion that the role of attention is to weight the precision of prediction errors [19]. However, given that the Posner paradigm compares expected, attended events to unexpected, unattended ones, applying it to these models does not provide insight into the potentially separable contributions of expectation and attention. By unconfounding these factors, our study lends credibility to the idea that expectation and attention interact in sensory processing.

In summary, we provide evidence that expectation might facilitate the withdrawing of resources from events by preparing to attenuate neural activity prior to their onset, but at the same time does not modulate pre-stimulus processing during attended time windows. This finding is of importance to studies on sensory processing which manipulate expectation and attention, whether jointly or in isolation.
